# Glucocorticoid receptor GRβ regulates glucocorticoid-induced ocular hypertension in mice

**DOI:** 10.1038/s41598-018-19262-9

**Published:** 2018-01-16

**Authors:** Gaurang C. Patel, Yang Liu, J. Cameron Millar, Abbot F. Clark

**Affiliations:** North Texas Eye Research Institute, University of North Texas Health Science Center, Fort Worth, TX 76107 United States

## Abstract

Prolonged glucocorticoid (GC) therapy can cause GC-induced ocular hypertension (OHT), which if left untreated progresses to iatrogenic glaucoma and permanent vision loss. The alternatively spliced isoform of glucocorticoid receptor GRβ acts as dominant negative regulator of GR activity, and it has been shown that overexpressing GRβ in trabecular meshwork (TM) cells inhibits GC-induced glaucomatous damage in TM cells. The purpose of this study was to use viral vectors to selectively overexpress the GRβ isoform in the TM of mouse eyes treated with GCs, to precisely dissect the role of GRβ in regulating steroid responsiveness. We show that overexpression of GRβ inhibits GC effects on MTM cells *in vitro* and GC-induced OHT in mouse eyes *in vivo*. Ad5 mediated GRβ overexpression reduced the GC induction of fibronectin, collagen 1, and myocilin in TM of mouse eyes both *in vitro* and *in vivo*. GRβ also reversed DEX-Ac induced IOP elevation, which correlated with increased conventional aqueous humor outflow facility. Thus, GRβ overexpression reduces effects caused by GCs and makes cells more resistant to GC treatment. In conclusion, our current work provides the first evidence of the *in vivo* physiological role of GRβ in regulating GC-OHT and GC-mediated gene expression in the TM.

## Introduction

Since their discovery in 1950’s, glucocorticoids (GCs) are the most widely prescribed medications worldwide because of their broad spectrum of anti-inflammatory and immunomodulatory activities^1,2^. Therapeutic GCs are prescribed to approximately 1.2% of US^3^ and 0.85% of UK^4^ populations, and the estimated worldwide use of GCs to be more than $10 billion per year^5^. GCs also remain the mainstay of treatment for a variety of ocular inflammatory diseases including uveitis^6–8^, macular degeneration^9^, and diabetic retinopathy^10,11^. They are also widely used to treat and prevent corneal transplant rejection^12,13^. The most common routes of GC administration in the treatment of these ocular disorders are topical ocular and/or intravitreal injections and implants^1,14^, although oral, systemic, and periocular injections (including subconjunctival, subtenon, retrobulbar, and peribulbar) routes of ocular delivery are also used^15^. Unfortunately, the therapeutic benefits of long term GC therapy are limited by serious local and systemic side effects. The serious ocular side effects of prolonged GC therapy include the development posterior subcapsular cataracts, significant GC-induced elevation in IOP and the development of GC-induced ocular hypertension (GC-OHT), and iatrogenic open-angle glaucoma^16–18^.

Over the past 50 years, there has been a suggested link between primary open angle glaucoma (POAG) and GC-induced glaucoma^16–20^. The development of GC-induced OHT depends on GC dose and duration of treatment, the method of administration, GC potency, as well as individual susceptibility to GCs^18,19^. Along with side effects of GC therapy, differences in individual ocular responsiveness to GCs (i.e. development of GC-OHT) is an important challenge in the therapeutic application of GCs; 90% of glaucoma patients are steroid responders compared to 40% of the general population^16,18,19,21,22^. In GC-induced OHT, GC affects the trabecular meshwork (TM), a small filter like tissue at the iris corneal junction that maintains normal intraocular pressure (IOP) by regulating aqueous humor outflow resistance. GCs alter TM cellular structure and functions, including increasing TM cell and nucleus size^17^, reorganizing the cytoskeleton (forming cross-linked actin networks (CLANs))^23–27^, inhibiting phagocytosis^28^, inhibiting cell proliferation and migration, altering cellular junctional complexes^29^, and increasing extracellular matrix deposition^30–34^. These biochemical and morphological changes in the TM affect TM stiffness and impair TM functions, causing increased aqueous humor outflow resistance and elevated IOP, clinically similar to what is observed in POAG patients. However, the exact molecular mechanisms responsible for steroid responsiveness and GC-induced OHT are not entirely clear.

GC effects are mediated by the glucocorticoid receptor isoform alpha (GRα), a ligand activated transcription factor and a member of nuclear receptor superfamily. The GRα has three domains: an N-terminal transactivation domain (NTD), a central DNA-binding domain (DBD), and a C-terminal ligand-binding domain (LBD)^35^. Human GR is encoded by the *NR3C1* gene that consists of nine exons^36,37^. The GR protein coding region is formed by exons 2–9, whereas exon 1 encodes the 5′-untranslated region. Exon 2 forms the N-terminal domain of GR, exons 3 to 4 constitute the central DBD, and exons 5 to 9 encode the hinge and LBD^5^. Heterogeneity in the GR results from alternative splicing of GR isoforms. GRα and GRβ are the two major splice variants resulting from alternative splicing at exon 9. Amino acid sequence analysis revealed that GRα and GRβ isoforms are identical from the amino terminus to amino acid 727 but diverge beyond this position, with GRα having an additional 50 amino acids and GRβ having an additional, non homologous 15 amino acids^38,39^. Both GRα and GRβ are expressed in cultured human TM cells^40–42^. GRα is the classical GR isoform that is responsible for most of the physiological and pharmacological effects of GCs^39,43^. Due to differences in the C-terminal domain, GRβ does not bind to GC ligands, resides primarily in nucleus, and does not activate GC responsive genes^44,45^. The GRβ isoform acts as a natural dominant negative inhibitor of GRα-induced transactivation of glucocorticoid-responsive genes^38,43^. GRβ antagonizes GRα activities by competition for GC response elements, by forming heterodimers with GRα inhibiting its transcriptional activities, and by interfering with coregulators that form transcriptional complexes on target genes^17,46^.

The relative expression levels of GRα and GRβ regulate GC sensitivity and specificity in various cells and tissues. This is supported by reports showing increased expression of GRβ in GC-resistant diseases^44,47^. Previous work in our laboratory found that GRα to GRβ ratios regulate GC responsiveness in TM cells and that glaucomatous TM (GTM) cells express lower GRβ and therefore are more susceptible to GCs^40,48^. We have also shown that selective mRNA splicing modulators (spliceosome proteins and thailanstatins) that specifically increase alternative splicing of GRβ are protective and inhibit dexamethasone (DEX) induced changes in TM cells^49,50^. Overexpression of GRβ in TM cells also inhibits DEX induced suppression phagocytic activity^28^. This suggests the potential role of GRβ in regulating steroid responsiveness in the TM. Thus, manipulating GRα to GRβ expression ratios holds promise for desensitizing cells and tissues to deleterious GC effects.

The goal of our study was to use viral vectors to selectively overexpress the hGRβ isoform in the TM of mouse eyes treated with GCs, to precisely dissect the role of alternatively spliced isoform GRβ in regulating steroid responsiveness. We hypothesized that overexpression of GRβ in the TM will reduce DEX- induced ocular hypertension, reduce GC-mediated biochemical and morphological changes in the TM, and lead to GC resistance in the TM upon GC treatment. We used an adenoviral expression vector serotype (Ad5) that selectively transduces the TM^51–55^ to overexpress the hGRβ isoform using our newly developed mouse model of DEX- induced OHT^56^. This reproducible model is easy to run and captures many aspects of GC-induced OHT observed in humans including elevated IOP, reduction in the aqueous humor outflow facility, biochemical changes in TM, and reversibility of ocular hypertension after discontinuing GC treatment^56^.

## Results

### Characterization of MTM cells from C57BL/6J mice

TM cells are actively phagocytic *in vitro* and *in vivo* to clear debris and pigment granules in aqueous humor^57–60^. We took advantage of this activity of TM cells to isolate the MTM cells from C57BL/6 J mice. We injected magnetic beads into the anterior chamber as described previously^61^. The beads were phagocytized by MTM cells and then MTM cells with engulfed magnetic beads were separated from non-TM cells by applying a magnetic field. Once in culture, a number of criteria were used to characterize these as TM cells including the expression of Col IV, laminin, α-smooth muscle actin (α-SMA) (Figure S1), as well as DEX-induced gene expression changes. DEX treatment increased the mRNA expression of MYOC, Collagen IV, and Elastin (Figure S2) compared with control and vehicle (ethanol) treated groups as shown by qPCR.

### GRβ overexpression in MTM cells inhibits DEX induced changes *in vitro*

**Figure 1:**
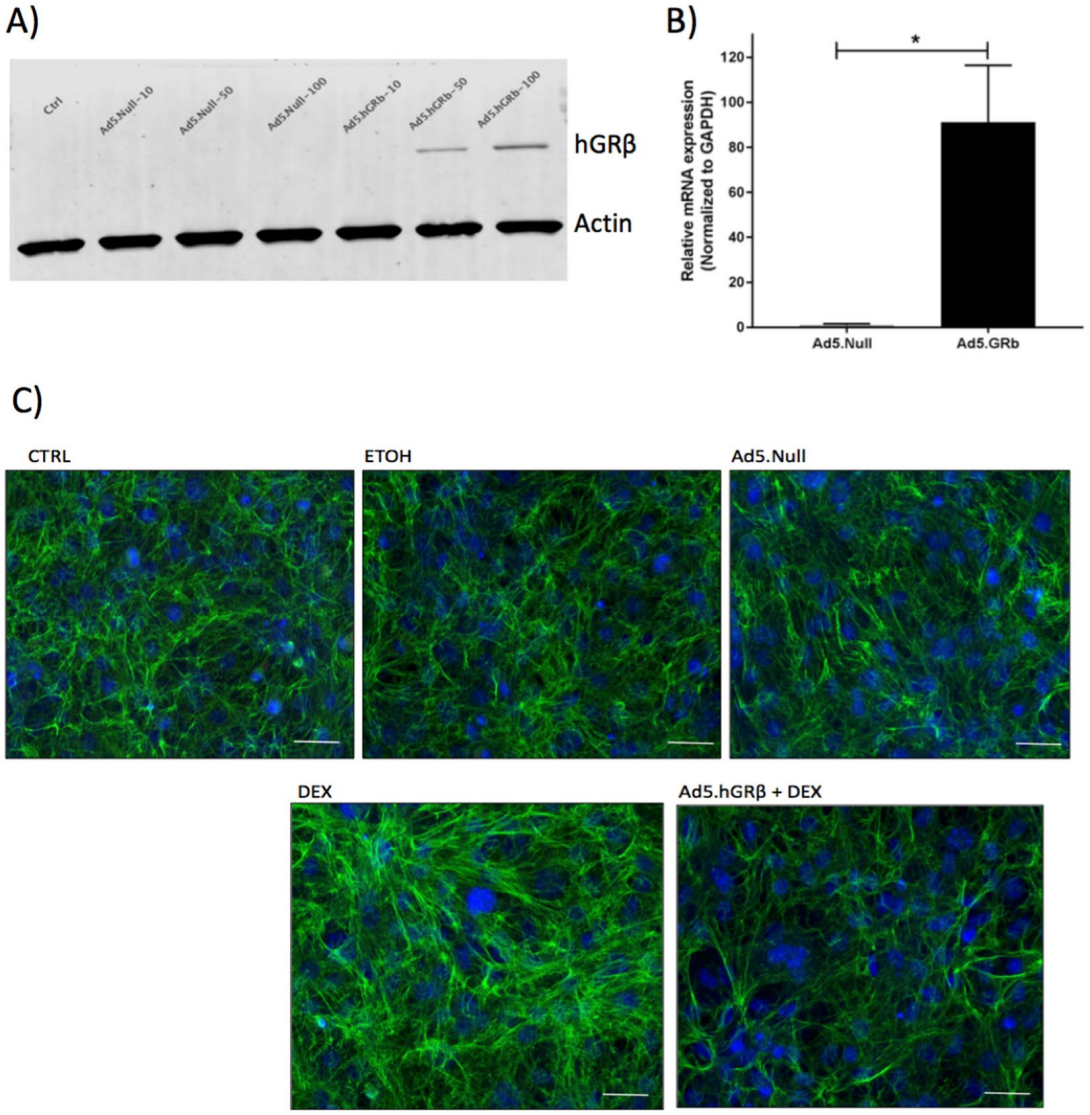
Inhibition of DEX induced Fibronectin (FN) expression by hGRβ overexpression in cultured MTM cells. Transduction of MTM cells with Ad5 vectors carrying null or hGRβ at different MOIs was detected by hGRβ western immunoblot and at mRNA levels. (**A**) hGRβ overexpression was detected at MOIs 50 and 100 in western immunoblotting. (**B**) qPCR showed increased expression of hGRβ; data are presented as means ± SEM, *P = 0.02, n = 3, unpaired student’s t-test. DEX treatment of MTM cells increased FN (**C**) expression, whereas transduction of MTM cells with Ad5.hGRβ inhibited DEX-induced FN expression as shown by immunocytochemistry. Control (CTRL), Ethanol (ETOH) and Ad5.Null treatments served as controls for these experiments. Green color represents FN staining (in Fig. 1B). Blue color represents DAPI staining showing cell nuclei. Actin served as loading control in Fig. 1A. 20× magnification. Representative data for 3 experimental triplicates.

Adenoviral vectors carrying hGRβ (Ad5.hGRβ) and Ad5.null were transduced in cultured MTM cells at different multiplicity of infections (MOI) to check for transduction efficiency at the protein level. We found that at MOIs 50 and 100, MTM cells were sufficiently overexpressing hGRβ as detected by western immunoblotting (Fig. 1A). In addition, we also found efficient transduction of Ad5.hGRβ in MTM cells at mRNA levels using qPCR (Fig. 1B). Next, MTM cells were transduced at MOI-50 followed by DEX treatment. DEX treatment increased fibronectin expression in MTM cells, which was inhibited by GRβ overexpression (Fig. 1C).

### GRβ overexpression in mouse trabecular meshwork

**Figure 2:**
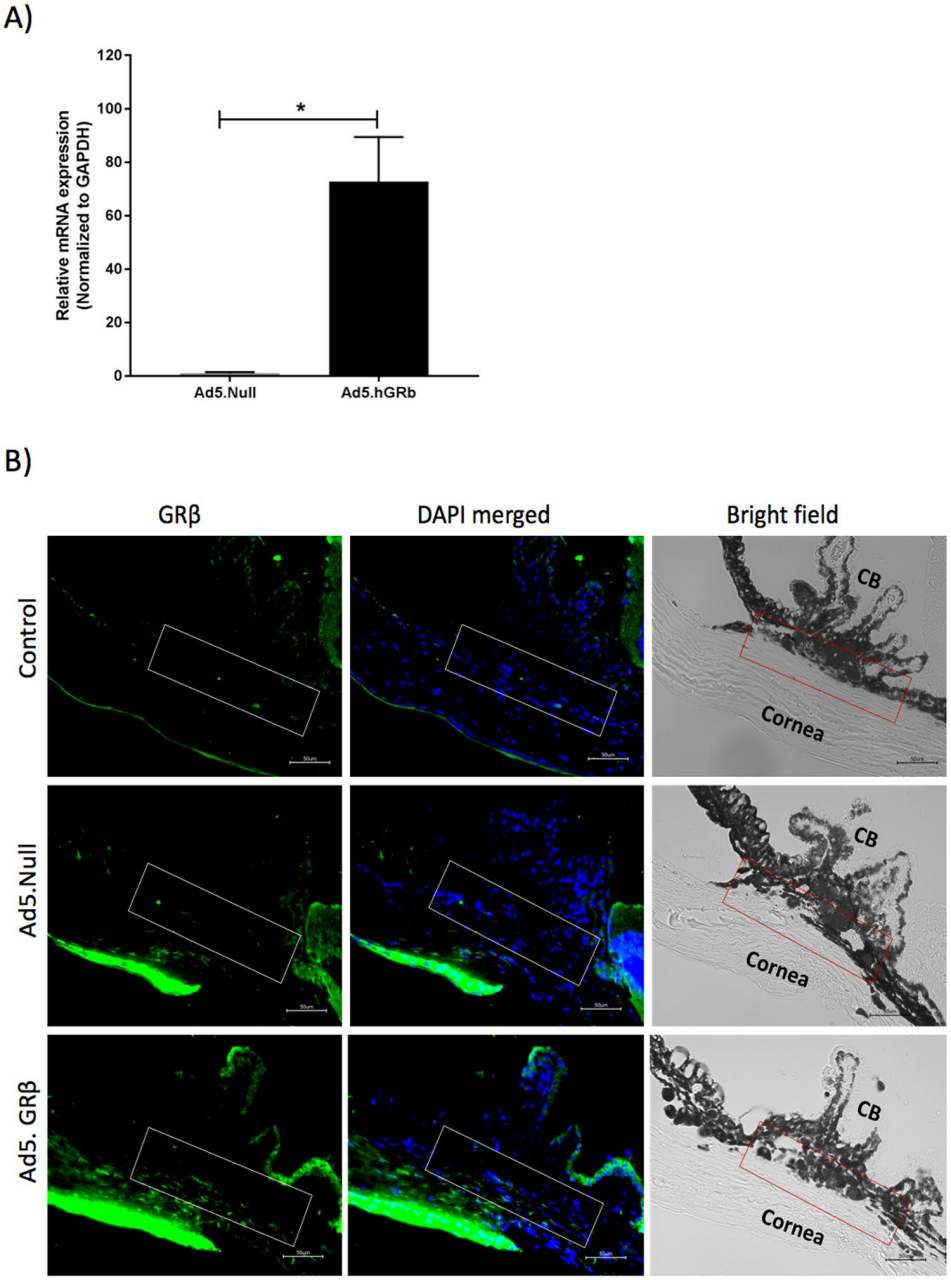
Overexpression of Ad5.hGRβ in mouse trabecular meshwork. (**A**) qPCR showing increased expression of hGRβ in anterior segment of mouse eyes; data are presented as means ± SEM, *P = 0.01, n = 3, unpaired student’s t-test. (**B**) Immunohistochemical analysis showing increased hGRβ (green) expression in the TM of Ad5.hGRβ (n = 3) treated mice compared to control (n = 2) and Ad5.Null (n = 3) treated mice. DAPI staining (blue) counterstains cell nuclei. Bright field image showing structural orientation of TM with respect to other ocular structures. White and red rectangular box shows TM. Scale bar: 50 μm.

To assess whether adenoviral viral vectors (Ad5) can selectively overexpress GRβ in the TM, adenoviral vectors encoding hGRβ (Ad5.hGRβ) and Ad5.null were intravitreally injected in mouse eyes. We found increased expression of hGRβ in anterior segment tissue by qPCR (Fig. 2A). Immunohistochemical analysis revealed increased expression of hGRβ in the TM of Ad5.hGRβ treated mice (n = 3) compared to uninjected control (n = 2) and Ad5.Null (n = 3) treated mice (Fig. 2B). Bright field images revealed no apparent ocular abnormalities and similar TM structural organization between control, Ad5.Null, and Ad5.hGRβ treated mice (Fig. 2B). In addition, adenoviral viral vector (Ad5) transduction did not induce apparent inflammation as shown by no changes in the expression of inflammatory cytokines (IL-6, TNF-α, IL-8, and IL-1α) between Ad5.hGRβ and Ad5.null treated groups in MTM cells and anterior segment tissue lysates (Figure S3).

### GRβ overexpression inhibits DEX-Ac induced IOP elevation

**Figure 3:**
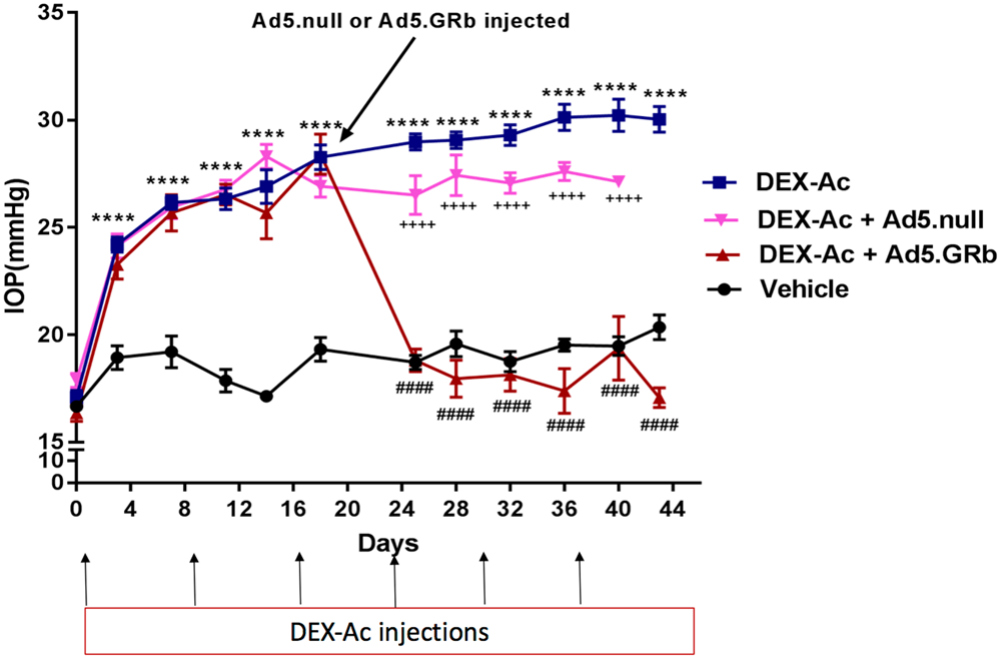
Inhibition of DEX-Ac induced OHT in mice after Ad5.hGRβ overexpression. Weekly periocular CF injections of DEX-Ac in both eyes significantly elevated IOP in mice. Nighttime IOP measurements were performed between different treatment groups of mice- DEX-Ac (n = 18), Vehicle (n = 10), DEX-Ac + Ad5.hGRβ (n = 18) and DEX-Ac + Ad5.null (n = 12). GRβ transduction of the TM at day 18 after DEX-Ac induced IOP elevation significantly lowered IOP within 7 days to baseline IOPs and remained at baseline throughout the end of the study, thus reversing GC-OHT in mouse eyes. Data are presented as means ± SEM. ^####^P < 0.0001 (DEX-Ac versus Ad5.hGRβ), ^++++^P < 0.0001 (DEX-Ac + Ad5.hGRβ versus DEX-Ac + Ad5.null), and ****P < 0.0001 (DEX-Ac versus vehicle and vehicle versus DEX-Ac, DEX-Ac + Ad5.hGRβ, DEX-Ac + Ad5.null until day 18), One-way ANOVA.

Mice were divided into the following treatment groups: a) Vehicle, b) DEX-Ac, c) DEX-Ac + Ad5.hGRβ, and d) DEX-Ac + Ad5.null. Weekly periocular CF injections of DEX-Ac suspension to both eyes caused DEX-induced OHT with sustained and significantly elevated IOP. Nighttime IOP elevation was rapid and significantly higher in DEX-Ac-treated mice compared with vehicle-treated mice starting 3-days post-injection. IOP differences between DEX-Ac and vehicle-treated mice in groups (a) and (b) were significantly higher throughout the study (Fig. 3, Table 1; *P* < 0.001). The absolute increases in IOP in DEX-Ac (n = 18) versus vehicle (n = 10) treated mice averaged 6.9 ± 0.9 mmHg at day 7, 9.7 ± 0.8 mmHg at day 14, 10.25 ± 0.7 mmHg at day 25, 10.6 ± 1.2 mmHg at day 36, and 9.67 ± 0.7 mmHg at day 43 (Mean ± SEM). In the other DEX treatment groups, IOP elevation also was significant and sustained compared to vehicle treated mice. At day 18 adenoviral vectors were injected (Fig. 3, Table 1).

**Table 1:** Comparison of IOPs between different treatment groups.

Comparison	Days	IOP differences between Means (mmHg) ± SEM	P-value (As indicated by 1-Way ANOVA followed by Dunnett posthoc test)	Significance signs in Fig. 2
Vehicle (n = 10) v/s DEX-Ac (n = 18)	0	0.08 ± 0.0.5	P > 0.05	N.S
	3	5.2 ± 0.9	P < 0.001	****
	7	6.9 ± 0.9	P < 0.001	****
	11	8.4 ± 0.7	P < 0.001	****
	14	9.7 ± 0.8	P < 0.001	****
	18	8.9 ± 0.9	P < 0.001	****
	25	10.25 ± 0.7	P < 0.001	****
	28	9.4 ± 0.9	P < 0.001	****
	32	10.54 ± 0.9	P < 0.001	****
	36	10.6 ± 1.2	P < 0.001	****
	40	10.74 ± 0.9	P < 0.001	****
	43	9.67 ± 0.7	P < 0.001	****
Vehicle (n = 10) v/s DEX-Ac + Ad5.hGRβ (n = 18)	0	0.7 ± 0.4	P > 0.05	N.S
	3	4.3 ± 0.8	P < 0.001	****
	7	6.4 ± 0.9	P < 0.001	****
	11	8.6 ± 0.6	P < 0.001	****
	14	8.5 ± 1.08	P < 0.001	****
	18	9 ± 1.0	P < 0.001	****
Vehicle (n = 10) v/s DEX-Ac + Ad5.Null (n = 12)	0	0.8 ± 0.5	P > 0.05	N.S
	3	5.1 ± 0.7	P < 0.001	****
	7	6.7 ± 1.0	P < 0.001	****
	11	8.8 ± 0.7	P < 0.001	****
	14	11.15 ± 0.7	P < 0.001	****
	18	7.5 ± 1.0	P < 0.001	****
**After Adenoviral injections at Day 18**				
DEX-Ac (n = 18) v/s DEX-Ac + Ad5.hGRβ (n = 18)	25	10.16 ± 0.6	P < 0.001	####
	28	11.1 ± 0.87	P < 0.001	####
	32	11.16 ± 0.8	P < 0.001	####
	36	12.73 ± 1.1	P < 0.001	####
	40	10.85 ± 1.2	P < 0.001	####
	43	12.97 ± 1.0	P < 0.001	####
DEX-Ac (n = 18) v/s DEX-Ac + Ad5.Null (n = 12)	25	2.4 ± 0.7	P < 0.05	
	28	1.6 ± 1.0	P > 0.05	N.S
	32	2.2 ± 0.9	P < 0.05	N.S
	36	2.5 ± 1.2	P > 0.05	N.S
	40	3.0 ± 1.8	P > 0.05	N.S
DEX-Ac + Ad5.Null (n = 12) v/s DEX-Ac + Ad5.hGRβ (n = 18)	25	7.6 ± 0.8	P < 0.001	++++
	28	9.4 ± 1.0	P < 0.001	++++
	32	8.9 ± 0.8	P < 0.001	++++
	36	10.22 ± 1.1	P < 0.001	++++
	40	7.7 ± 2.0	P < 0.001	++++

Adenoviral vectors carrying Ad5.hGRβ and Ad5.null were intravitreally injected in the DEX-Ac + Ad5.hGRβ and DEX-Ac + Ad5.null groups, respectively, and IOP measurements continued to be recorded. In the DEX-Ac + Ad5.hGRβ treatment group, IOP was significantly reduced and returned to baseline IOP within one week as compared with DEX-Ac and DEX-Ac + Ad5.null treatment groups. The IOP remained at baseline throughout the study even though these mice continued to receive weekly DEX-Ac treatment. After GRβ overexpression, there was a 10–13 mmHg decrease in IOP, thus totally inhibiting DEX-Ac induced OHT in mice (Fig. 3, Table 1; *P* < 0.001). The absolute decrease in IOP in DEX-Ac (n = 18) versus DEX-Ac + Ad5.hGRβ (n = 18) treated mice averaged 10.16 ± 0.6 mmHg at day 25, 11.1 ± 0.87 mmHg at day 28, 11.16 ± 0.8 mmHg at day 32, 12.73 ± 1.1 mmHg at day 36, 10.85 ± 1.2 mmHg at day 40, and 12.97 ± 1.0 mmHg at day 43 (Mean ± SEM).

Ad5.Null vectors alone did not alter IOP, while IOP elevation was significant and sustained with DEX-Ac + Ad5.null treatment throughout the study. In addition, there were significant differences in IOP between DEX-Ac + Ad5.hGRβ and DEX-Ac + Ad5.null (Fig. 3, Table 1).

### GRβ overexpression returns conventional outflow facility (C) to normal levels

**Figure 4:**
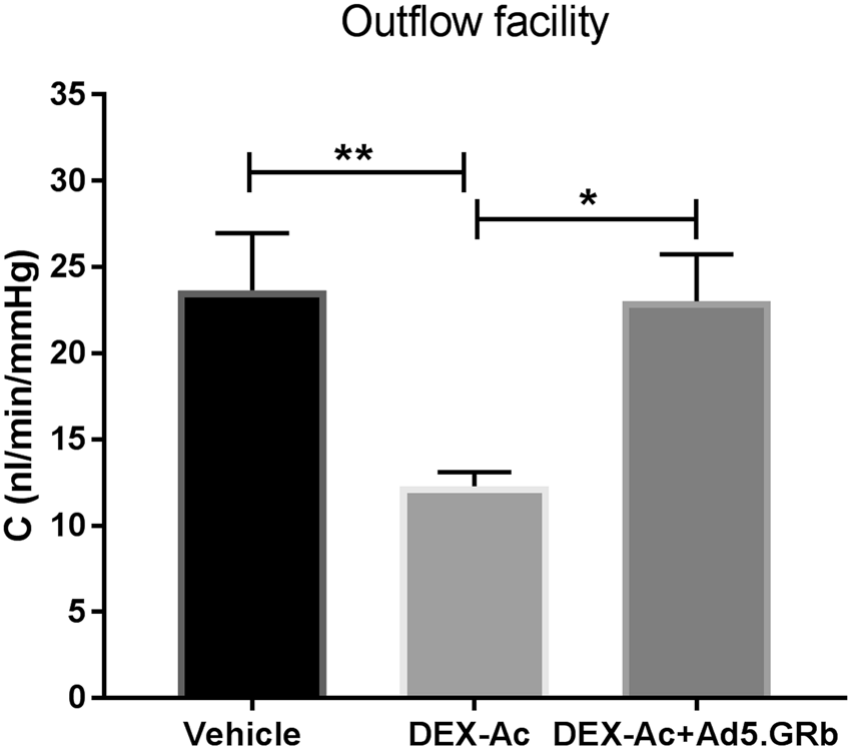
Comparison of conventional outflow facility (C) between DEX-Ac, vehicle, and DEX-Ac + Ad5.hGRβ-treated mice. After 6 weeks of treatment, DEX-Ac (n = 14) significantly decreased the C compared to vehicle (n = 13) treated mice. GRβ transduction (DEX-Ac + Ad5.hGRβ; n = 13) returned the outflow facility to normal levels compared to DEX-Ac treated mice. Data are presented as means ± SEM. *P < 0.05, **P < 0.01. One-Way ANOVA.

Conventional outflow facility (C) was measured in live mice after six weeks of treatment. C was significantly decreased in DEX-Ac mice compared to vehicle-treated mice. However, DEX-Ac + Ad5.hGRβ treatment restored the C to normal levels compared to DEX-Ac treated mice (Fig. 4). C was 12.29 ± 0.8 nL/min/mmHg in DEX-Ac treated mice (n = 14) compared 23 ± 2.7 nL/min/mmHg in DEX-Ac + Ad5.hGRβ and 23.62 ± 3.3 nL/min/mmHg in vehicle-treated mice (n = 13). The mean increases in outflow facility corresponded well with the mean IOP decrease in DEX-Ac + Ad5.hGRβ treated mice according to the modified Goldman equation: IOP = [(Fin − Fu)/C] + Pe. In this equation, Fin represents aqueous humor production rate (uL/min), C represents trabecular outflow facility (uL/min/mmHg), Fu represents uveoscleral outflow rate (uL/min), and Pe represents episcleral venous pressure (mmHg).

### Biochemical changes in TM after GRβ overexpression

**Figure 5:**
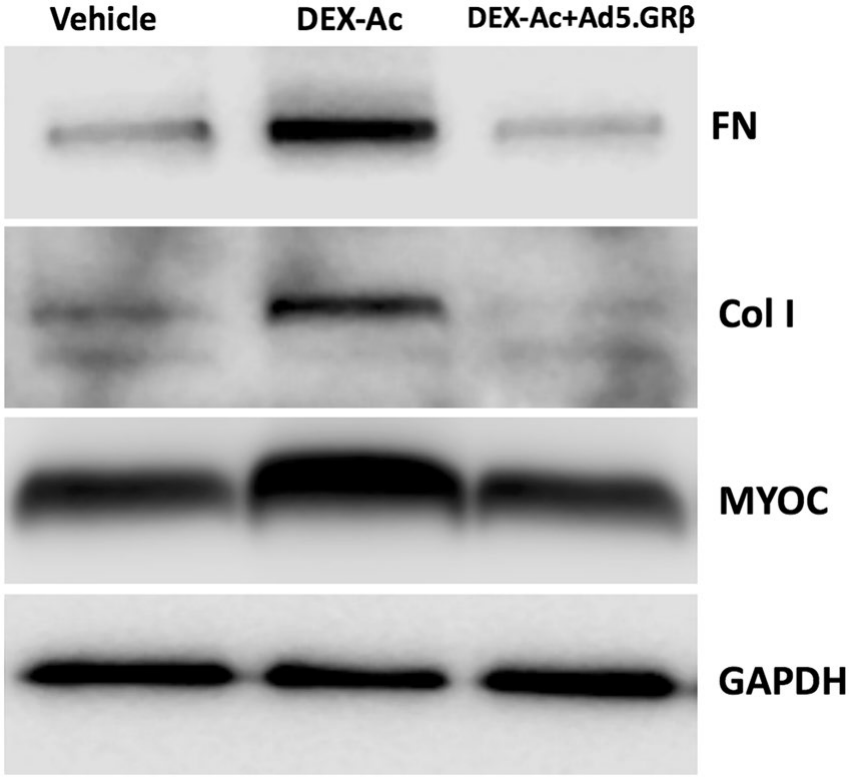
DEX-Ac + Ad5.hGRβ treatment decreased expression of fibronectin (FN), collagen I (Col I), and myocilin (MYOC) in the anterior segment of the mouse eyes. Representative western blot image of fibronectin (FN), collagen I (Col I), and myocilin (MYOC) in the anterior segment tissue lysates of DEX-Ac, vehicle, and DEX-Ac + Ad5.hGRβ treated mice. DEX-Ac treatment increased FN, Col I, and MYOC expression, which returned to normal control levels in DEX-Ac + Ad5.hGRβ treated mice. GAPDH served as loading control. Representative data from following anterior segments per group: DEX-Ac n = 3; DEX-Ac + Ad5.hGRβ n = 3; vehicle n = 2. Please refer Supplementary Figures 4, 5, 6, and 7 for full-length blots as representative image is cropped.

DEX treatment leads to many biochemical changes in the TM, including increased production of fibronectin^31^, collagens^33^, and myocilin^56^. To assess whether treatment with Ad5.hGRβ will also inhibit these DEX-induced biochemical changes, we performed western immunoblot analysis for fibronectin, collagen I, and myocilin (MYOC) in anterior segment tissues from 6-week DEX-Ac, DEX-Ac + Ad5.hGRβ, and vehicle-treated mice (Fig. 5). Western blot analysis revealed increased fibronectin, collagen I, and MYOC expression in the TM of DEX-Ac treated mice compared to DEX-Ac + Ad5.hGRβ and vehicle-treated mice. DEX-Ac + Ad5.hGRβ reduced these biochemical changes in the TM. (DEX-Ac n = 3; DEX-Ac + Ad5.hGRβ n = 3; vehicle n = 2). Full length blots for all western blot analyzed proteins are attached as supplementary files (Supplementary Figures 4, 5, 6 and 7).

## Discussion

**Figure 6:**
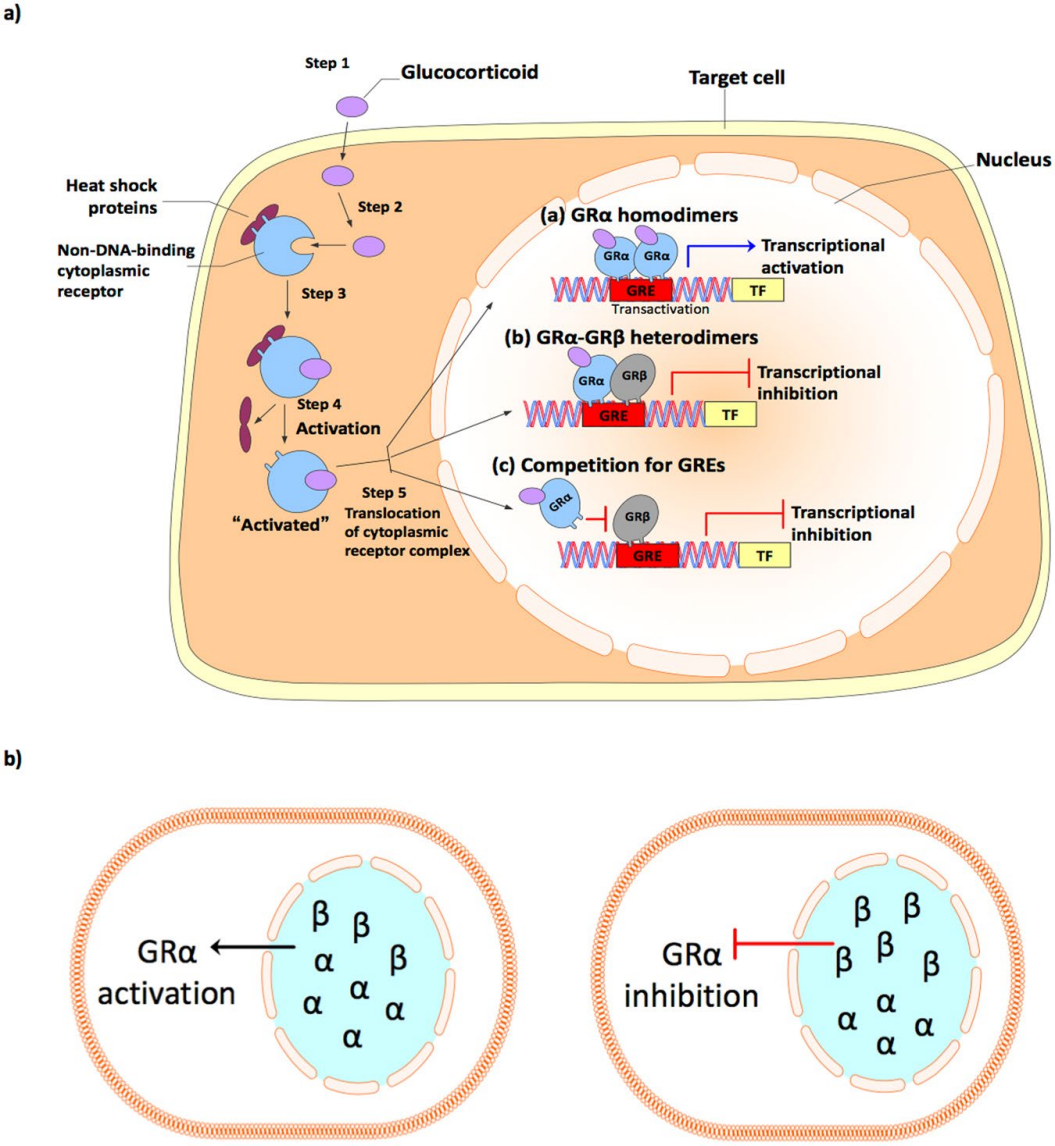
Mechanism of action of GRβ. (**a**) The classical pharmacological and physiological actions of GCs are mediated by the GR isoform GRα. In the absence of ligand (i.e GCs), GRα predominantly resides in the cytoplasm of cells as part of a large multiprotein complex that includes chaperone proteins (hsp90, hsp70, and p23) and immunophilins (FKBP51 and FKBP52), maintaining the high-affinity ligand binding GR confirmation. Upon binding ligand, GRα undergoes a conformational change, resulting in the dissociation of the multiprotein complex (Steps 1, 2, and 3). Structural reorganization of the GRα protein exposes nuclear localization signals, and the ligand-bound GRα is rapidly translocated by microtubule motor proteins along the microtubules into the nucleus through nuclear pores (Steps 4 and 5). Once inside the nucleus, GRα forms homodimers that bind directly to GREs and stimulate target gene expression (**a**). The GRβ isoform acts as a natural dominant negative inhibitor of GRα-induced transactivation of glucocorticoid-responsive genes. GRβ antagonizes GRα activities by forming heterodimers with GRα inhibiting its transcriptional activities (**b**), and by competition for GC response elements (c). (**b**) GRβ overexpression in the cell leads to GRα inhibition and make cells more resistance to GCs.

The clinical management of GC-OHT in patients undergoing prolonged GC therapy requires monitoring and lowering IOP with glaucoma drugs and/or surgery^18,19^. In addition, if left untreated, GC-OHT progresses to secondary open angle glaucoma, causing glaucomatous optic neuropathy and permanent vision loss. Another important challenge in the therapeutic application of GCs is heterogeneity in individual responsiveness to GC treatment^16,21,22^. A better understanding of the molecular mechanisms of steroid responsiveness and the etiology of GC-induced OHT and glaucoma will lead to better therapeutic options. The alternatively spliced isoform of the glucocorticoid receptor GRβ acts as dominant negative regulator of GRα activity^38,43–45^. A GRβ gene therapy approach based our current molecular findings would offer new options for the management of GC-OHT and glaucoma. In our study, we show that overexpression of GRβ inhibits GC effects on MTM cells *in vitro* and GC-induced OHT in mouse eyes *in vivo*. Ad5 mediated GRβ overexpression reduced the GC induction of fibronectin, collagen 1, and myocilin in TM of mouse eyes both *in vitro* and *in vivo*. GRβ also reversed DEX-Ac induced IOP elevation, which correlated with increased conventional aqueous humor outflow facility. Thus, GRβ overexpression reduces effects caused by GCs on the TM and makes cells more resistance to GC treatment (Fig. 6b). These results are consistent with our previous studies showing that GRβ regulates GC responsiveness in human TM cells and that overexpressing GRβ inhibits GC-induced and glaucomatous damage in HTM cells^28,40,48–50,62^.

Furthermore, animal models of GC induced OHT have been reported in many species in addition to man^63^. We selected mouse as the model for our study based on our previous work showing reproducible DEX- induced OHT, with no obvious systemic side effects^56^. The structural and functional anatomy of the trabecular outflow pathway in the mouse is similar to humans, including a lamellar TM and canal of Schlemn. Mice, like humans, lack the washout effect that is observed in almost all other non-human eyes^64^. Using mice also allows smaller amounts of test compounds and adenoviral vectors for transduction of the TM^51^. Furthermore, the entire mouse genome is known and easily manipulated genetically, thus allowing future studies including evaluation of the role of the *MYOC* glaucoma gene on DEX-OHT^56^. Other mouse models of GC-induced OHT^65–68^ have been reported, but these require systemic GC exposure or alter aqueous outflow without IOP elevation. Therefore we used our recently developed novel model of DEX-Ac induced OHT to evaluate the effect of GRβ overexpression because this model is easy to run, highly reproducible, and mimics many aspects of GC-induced OHT in humans^56^. Although we routinely use mice aged 6 to 8 months in our experiments, we also tested younger mice (aged 2 to 3 months) and obtained similar steroid response results. This finding also validates the results obtained *in vitro* with MTM cells isolated from younger mice. In addition, we also found that the sex of mice does not have a major role in development of DEX-Ac induced OHT^56^. We have previously published our protocol for measurement of dark adapted nocturnal IOP in mice^56,69^ and small dark adaption time does not appear to affect the circadian rhythm of mice. Several studies^70–72^ have shown that keeping mice in acute dark for 24–48 hours does not affect their circadian rhythm, which still follows the 24 hour biphasic IOP pattern (with low IOP during day and high during night). In contrast, exposure to constant light does change the circadian rhythm. In addition, another study^73^ suggested that the 24 hour IOP change pattern was not driven by light perception but is endogenously regulated by clock genes in the suprachiasmatic nucleus. Furthermore, Ding *et al*.^74^ studied effects of general anesthesia on IOP, and their data suggest that there is little effect on IOP measured using isoflurane, if the entire procedure is completed within 3–5 minutes of anesthesia. We completed our IOP measurements for both eyes within 3–5 minutes. In our model, we were able to achieve a DEX-mediated IOP elevation of 10–11 mmHg from baseline, and GRβ overexpression reversed this DEX-OHT by decreasing IOP ∼ 10–13 mmHg, which remained at baseline despite continued DEX-Ac administration. These findings were well correlated with conventional outflow facilities measured at the end of the experiment. DEX-Ac treatment significantly reduced outflow facility compared with vehicle treatment, which agrees with previous studies^56,65–67^. However, GRβ overexpression significantly increased the outflow facility, returning it to normal levels despite continued DEX-Ac treatment. Consistent with other previously reported studies^24,31,33,65,67^ that show DEX-induced biochemical changes in the TM, we found similar biochemical changes in TM of our mice treated with DEX-Ac. The expression of fibronectin, collagen I, and myocilin increased in the TM of DEX-Ac treated mice compared to vehicle-treated mice. However, GRβ overexpression reduced the expression of these DEX induced proteins in the TM. Thus, our mouse model is an important tool to study the *in vivo* function of GRβ in the TM during GC-OHT, and we have shown that GRβ overexpression inhibits DEX-Ac induced OHT in mice.

Adenoviral viral vectors (Ad5) were used in this study to selectively overexpress GRβ because they can transduce dividing as well as terminally differentiated cells, and the CMV promoter drives rapid and high expression of the encoded transgene. In addition, we and others have used intravitreal delivery of Ad5 vectors in mice to effectively and efficiently transduce the TM in order to study various glaucoma associated pathways^51–55^. Although Ad5 viral transduction of the gene of interest causes mild ocular inflammation^51,52^, we incorporated Ad5.Null as a relevant control treatment group. In addition, we also show that adenoviral viral vector (Ad5) transduction did not induce inflammation as shown by no change in expression of inflammatory cytokines (IL-6, TNF-α, IL-8, and IL-1α) in MTM cells and anterior segment tissue lysates (Figure S3). Moreover, Ad5 vectors are still widely used to establish proof of principle in ocular hypertension and glaucoma studies.

The mechanisms by which GRβ functions as a dominant negative regulator of GC responsiveness is depicted in Fig. 6. GRβ antagonizes GRα by competing with GC-responsive elements (GREs) on GC regulated genes. It also can form heterodimers with GRα bound to GREs, which inhibits transcription of downstream genes^17,46^. Thus, when we overexpress GRβ in TM of mouse eyes, GRβ inhibits GC-OHT in mice by one of these mechanisms. Interestingly, the GRβ isoform also is present in mice^75^, rat^76^ and zebrafish^77^ in addition to man. This alternative splicing to generate the GRβ isoform differs between species. In humans, this alternative splicing involves exons 9α and 9β, while in other species the splice site occurs within the intron separating exon 8 and 9. However, the GRβ isoforms between species are similar in structure and function to the human GRβ isoform. In addition, Hinds *et al*.^75^ have shown that mGRβ shares a greater than 87% sequence homology with hGRβ and shares similar properties with hGRβ, (i.e mGRβ does not bind to GC ligands, resides primarily in nucleus, does not activate GC responsive genes, and inhibits mGRα activity). Studies on resistance to GC therapy have reported elevated GRβ levels in several acute and chronic inflammatory diseases as well as depressive disorders^44,47^. Conversely, our lab has reported that TM cell strains from POAG patients have lower GRβ expression compared to TM cells derived from normal eyes, thereby making GTM cells more responsiveness to GC treatment^40,48^. This indicates that a lower GRβ to GRα ratio may contribute to greater sensitivity to GCs exhibited by POAG patients and a greater prevalence of GC-OHT in POAG compared to normals. Thus, it is possible to manipulate GRα to GRβ expression ratios in cells and tissues to desensitize them to GC effects. In our study, we used gene therapy to selectively overexpress GRβ in the TM so that GRβ will inhibit GRα transcriptional activities (Fig. 6b), which prevented GC-induced biochemical, morphological, and physiological (OHT) changes in TM.

In summary, our current work provides the first evidence of the *in vivo* physiological role of GRβ in regulating GC-OHT and GC-mediated gene expression in the TM. GRβ overexpression inhibits GC-OHT in mice by reducing elevated IOP, increasing C and reducing GC-mediated biochemical changes in the TM.

## Materials and Methods

### Animals

2–3 month old and retired breeder male and female C57BL/6 J mice were obtained from the Jackson Laboratory (Bar Harbor, ME). 2–3 month old mice were used for mouse trabecular meshwork (MTM) cell isolation and culture. Retired breeder mice 6 to 8 months old were used for all *in vivo* experiments. All mouse studies, care, and experiments were performed in compliance with the ARVO Statement of the Use of Animals in Ophthalmic and Vision Research and the University of North Texas Health Science Center Institutional Animal Care and Use Committee regulations. All experimental protocols were approved by the University of North Texas Health Science Center Institutional Animal Care and Use Committee. Mice were housed under controlled conditions of temperature (21 to 26 °C), humidity (40% to 70%) and a 12 hr light/12 dark cycle (with lights on at 7am and off at 7 pm). Food and water were provided *ad libitum*. The number of animals used in each experiment is indicated in the corresponding figure legends and results section.

### Dexamethasone acetate formulation and periocular conjunctival fornix injection

Dexamethasone acetate (DEX-Ac) 10 mg/ml and vehicle suspension were formulated as previously described^56^. To develop DEX-Ac induced OHT in mice, DEX-Ac was periocularly injected bilaterally using our previously described procedure^56^. Prior to and during injections, mice were anesthetized with isoflurane (2.5%) containing oxygen (0.8 L/min). For topical anesthesia, both eyes received 1 to 2 drops of 0.5% proparacaine HCl (Akorn Inc., Lake Forest, IL). The lower eyelid was retracted, and a 32-gauge needle with a Hamilton glass microsyringe (25 μL volume) (Hamilton Company, Reno, NV) was inserted through the conjunctival fornix (CF). DEX-Ac or vehicle suspension (20 μL) was injected immediately into the CF over the course of 10 to 15 seconds. The needle was then withdrawn. The procedure was performed on both eyes of each animal (each animal receiving either DEX-Ac OU or vehicle OU). Mice were treated with DEX-Ac or vehicle once per week until the end of the study.

### Adenoviral vectors injection

The Ad5 vectors were prepared by the Gene Transfer Core Facility at the University of Iowa (Iowa City, IA). Adenoviral vectors (Ad5) expressing human GRβ (Ad5.CMV.hGRβ) (University of Iowa, Iowa City, IA, USA) were used to overexpress GRβ in the TM of mouse eyes. Adenovirus null vector (Ad5.CMV.Null) (University of Iowa, Iowa City, IA, USA) was used as a negative control. All adenoviral vectors were intravitreally injected into both mouse eyes using previously described procedure^56^ [3 × 10^7^ plaque-forming units (pfu)]. Prior to and during injections, mice were anesthetized with isoflurane (2.5%) containing oxygen (0.8 L/min). For topical anesthesia, both eyes received 1 to 2 drops of 0.5% proparacaine HCl (Akorn Inc., Lake Forest, IL). The eyes were proptosed and a 33-gauge needle attached to a glass microsyringe (10μL volume) (Hamilton Company, Reno, NV) was inserted through the equatorial sclera and inserted into the vitreous chamber at an angle of ~45° taking care to avoid touching the posterior part of the lens or the retina. Ad5.hGRβ or Ad5.null (2 μL) was injected into the vitreous over the course of 1 minute. The needle was then left in place for a further 30 seconds (to facilitate mixing), before being rapidly withdrawn.

### IOP Measurements

For this study, isoflurane anesthetized IOPs were measured at night. For nighttime IOP measurement, mice were first kept in dark at 4 PM on day of IOP measurement, and at 10 PM, anesthetized IOPs were measured twice a week in both eyes using a TonoLab rebound tonometer. The entire procedure was performed in a darkroom using dim red light. IOP measurements for both eyes were completed in 3–5 minutes. IOPs were measured before start of DEX-Ac injections and twice a week after DEX-Ac injections throughout the study. In addition, after completion of IOP measurement, mice were kept in dark until 7am next day (when the next circadian rhythm cycle starts).

### Aqueous Humor Outflow facility (C) Measurements

Aqueous Humor Outflow facility (C) was measured using our constant flow infusion technique in live mice as previously described^64,78^. Mice were anesthetized using a 100/10 mg/kg ketamine/xylazine cocktail. A quarter to half of this dose was administered for maintenance of anesthesia as necessary. One to two drops of proparacaine HCl (0.5%) (Akorn Inc., Lake Forest, IL) were applied topically to both eyes for corneal anesthesia. The anterior chambers of both eyes were cannulated using a 30-gauge needle inserted through the cornea 1 to 2 mm anterior to the limbus and pushed across the anterior chamber to a point in the chamber angle opposite to the point of cannulation, taking care not to touch the iris, anterior lens capsule epithelium, or corneal endothelium. Each cannulating needle was connected to a previously calibrated (sphygmomanometer; Diagnostix 700, Hauppage, NY) flow-through BLPR-2 pressure transducer (World Precision Instruments (WPI), Sarasota, FL) for continuous determination of pressure within the perfusion system. A drop of phosphate buffered saline (PBS) was also administered to each eye to prevent corneal drying. The opposing ends of the pressure transducer are connected via further tubing to a 50μL syringe loaded into a microdialysis infusion pump (SP101I Syringe Pump; WPI). The tubing, transducer, and syringe were all filled with sterile PBS solution (filtered through a 0.2 μm HT Tuffryn Membrane Acrodisc Syringe Filter, PALL Gelman Laboratory, Port Washington, NY). Signals from each pressure transducer were passed via a TBM4M Bridge Amplifier (WPI) and a Lab-Trax Analog-to-Digital Converter (WPI) to a computer for display on a virtual chart recorder (LabScribe2 software, WPI). Eyes were initially infused at a flow rate of 0.1 μL/min. When pressures stabilized within 10 to 30 minutes, pressure measurements were recorded over a 10 minute-period, and then flow rates were increased sequentially to 0.2, 0.3, 0.4, and 0.5 μL/min. Three stabilized pressures at 5-minute intervals at each flow rate were recorded. C in each eye of each animal was calculated as the reciprocal of the slope of a plot of mean stabilized pressure as ordinate against flow rate as abscissa.

### MTM Cell Culture

MTM cells were isolated from 2–3 month old C57BL/6 J mice, and characterized using previously developed methodology^61^. MTM cells were cultured and maintained in Dulbecco’s modified Eagle’s medium (DMEM) (Invitrogen-Gibco Life Technologies, Grand Island, NY, USA) supplemented with 10% fetal bovine serum (FBS; Atlas Biologicals, Fort Collins, CO, USA), penicillin (100 units/mL), streptomycin (0.1 mg/mL), and L-glutamine (0.292 mg/mL) (Thermo Fisher Scientific, Rockford, IL, USA).

### *In vitro* transduction of MTM cells using adenoviral vectors and DEX treatment

MTM cells were transduced with adenoviral vectors carrying hGRβ (Ad5.h GRβ) or null vector (Ad5.null) at different multiplicity of infections (MOI- 10,50,100) for 48 hours to check for transduction efficiency. We selected MOI-50 based on transduction efficiency to transduce MTM cells for 48 hours prior to treatment with or without DEX (100 nM) for another 48 hours. MTM cells were divided into following groups: a) Control- no treatment, b) Vehicle control (0.1% Ethanol) treatment, c) DEX (100 nM) treatment, d) Ad5.null vector treatment, and e) Ad5.hGRβ + DEX treatment.

### RNA Isolation and Quantitative PCR (qPCR)

RNA was isolated from MTM cells and mouse anterior segments that were transduced with adenoviral vectors carrying hGRβ or null vector (Ad5.hGRβ, Ad5.null). RNA was extracted using an RNA purification kit (RNeasy Mini Kit; Qiagen, Valencia, CA, USA) with DNase I treatment for 15 minutes. RNA was quantified using the NanoDrop 2000 (Thermo Fisher Scientific). RNA was reversed transcribed into cDNA using the iScript cDNA synthesis kit (Bio-Rad). Quantitative PCR was performed using the SSoAdvanced SYBR Green Supermix (Bio-Rad) in a total volume of 20 μL in a CFX96 thermocycler (Bio-Rad). The thermoprofile consisted of 40 cycles of 95 °C for 10 seconds, 58 °C for 30 seconds, followed by a dissociation curve. The primers sequences for hGRβ and GAPDH were: hGRβ forward primer (5′-GAACTGGCAGCGGTTTTATC-3′), hGRβ reverse primer (5′-TCAGATTAATGTGTGAGATGTGCTT-3′), GAPDH forward primer (5′- GGGAGCCAAAAGGGTCAT-3′), and GAPDH reverse primer (5′-TTCTAGACGGCAGGTCAGGT-3′).

### Immunocytochemistry

Cells were cultured on glass coverslips in 24-well plates. At the end of the experiment, cells were fixed in 4% paraformaldehyde (Electron Microscopy Sciences, Hatfield, PA) and kept at 4 °C for 30 minutes. After PBS washing, cells were incubated with 0.5% Triton X-100 (Fisher Scientific, Pittsburgh, PA) in PBS at room temperature for 30 minutes, and then blocked with PBS Superblock (Thermo Scientific, Rockford, IL). Cells were then immunolabelled with primary antibody: rabbit polyclonal fibronectin antibody (1:200, EMD Millipore, Billerica, MA, USA; catalog # AB1945) and incubated at 4 °C overnight. Cells incubated without primary antibody served as a negative control. Following the incubation, cells were washed three times with PBS and further incubated for 1.5 hours at room temperature with the secondary antibody (Alexa goat anti-rabbit 488; 1:500; Thermo Scientific, Rockford, IL). After PBS washing, glass coverslips with cells were then mounted on ProLong gold anti-fade reagent with DAPI (Invitrogen-Molecular Probes, Carlsbad, CA, USA). All images were taken with a Nikon Eclipse Ti-U microscope with Nuance imaging system. All antibodies used in this study were validated and characterized previously^56,79^.

### Immunohistochemistry

Eyes from control, Ad5.Null, and Ad5.hGRβ treated mice were enucleated and fixed overnight in freshly prepared 4% paraformaldehyde (PFA) in PBS. Afterwards, eyes were washed three times with PBS, dehydrated with ethanol, and embedded in paraffin. Samples were sectioned at 5 μm. For immunostaining, tissue sections were deparaffinized in xylene and rehydrated twice each with 100%, 95%, 70%, and 50% ethanol for 5 minutes. Tissue sections were blocked (10% Goat serum + 0.2% Triton-X 100) for 2 hrs in a dark and humid chamber. Tissue sections were then washed briefly with PBS and immunolabeled with rabbit polyclonal glucocorticoid receptor beta antibody (1:50; Thermo Fisher Scientific, Inc.; catalog # PA3–514) incubated overnight at 4 °C. Tissue sections incubated without primary antibody served as a negative control. Following the incubation, tissue sections were washed three times with PBS and further incubated for 1.5 hrs at room temperature with the appropriate secondary antibodies (Alexa Goat anti-rabbit 488; 1:500; Thermo Fisher Scientific, Inc.). Tissue sections were washed with PBS and then mounted on ProLong gold anti-fade reagent with DAPI (Invitrogen-Molecular Probes, Carlsbad, CA, USA). Images were captured by Keyence all-in-one fluorescence microscope (Itasca, IL).

### Western blot analysis

Mouse anterior segments were carefully dissected from enucleated eyes and lyzed in Lysis buffer (T-PER, Thermo Scientific, Rockford, IL) containing Halt protease inhibitor cooktail (1:100; Thermo Scientific, Rockford, IL). The protein samples were run on denaturing 12% polyacrylamide gels and transferred onto PVDF membranes. Blots were blocked with 10% nonfat dried milk for one hour and then incubated overnight with specific primary antibodies at 4 °C on a rotating shaker. The membranes were washed thrice with 1 × PBST and incubated with corresponding HRP-conjugated secondary antibody for 1.5 hours. The proteins were then visualized using enhanced chemiluminescence detection reagents (SuperSignal West Femto Maximum Sensitivity Substrate; Pierce Biotechnology). The primary antibodies used were: mouse monoclonal fibronectin antibody (1:1000, Santa Cruz Biotechnology, catalog # sc18825), mouse monoclonal myocilin antibody (1:1000, Abnova, catalog # H00004653-M01), rabbit polyclonal collagen I antibody (1:1000, Santa Cruz Biotechnology, catalog # sc20649), and glyceraldehyde-3-phosphate dehydrogenase (1:1000; Cell Signaling Technology; catalog # 3683). All antibodies used in this study were validated and characterized previously^56,79^.

### Statistics

Statistical analyses were performed using GraphPad Prism Version 7.0 (GraphPad Software, La Jolla, CA). Unpaired student’s *t*-test (2-tailed) was used to compare data between two groups. One-way ANOVA followed by Dunnett’s, or Tukey’s post-hoc analysis tests was used to calculate statistical significance for comparison among three or more groups. A p < 0.05 was considered statistically significant.

## Electronic supplementary material

Supplementary Data
