# Supplementary material for: Glucocorticoid receptor GRβ regulates glucocorticoid-induced ocular hypertension in mice
Source: Sci Rep. 2018 Jan 16;8:862. doi: 10.1038/s41598-018-19262-9 (PMC5770444; doi:10.1038/s41598-018-19262-9)
Supplement: Supplementary file 1 — Supplementary Data [file 41598_2018_19262_MOESM1_ESM.pdf]

Glucocorticoid receptor GRβ regulates glucocorticoid-induced ocular hypertension in mice

Gaurang C. Patel, Yang Liu, J. Cameron Millar, and Abbot F. Clark*

Supplementary Data

Figure S1

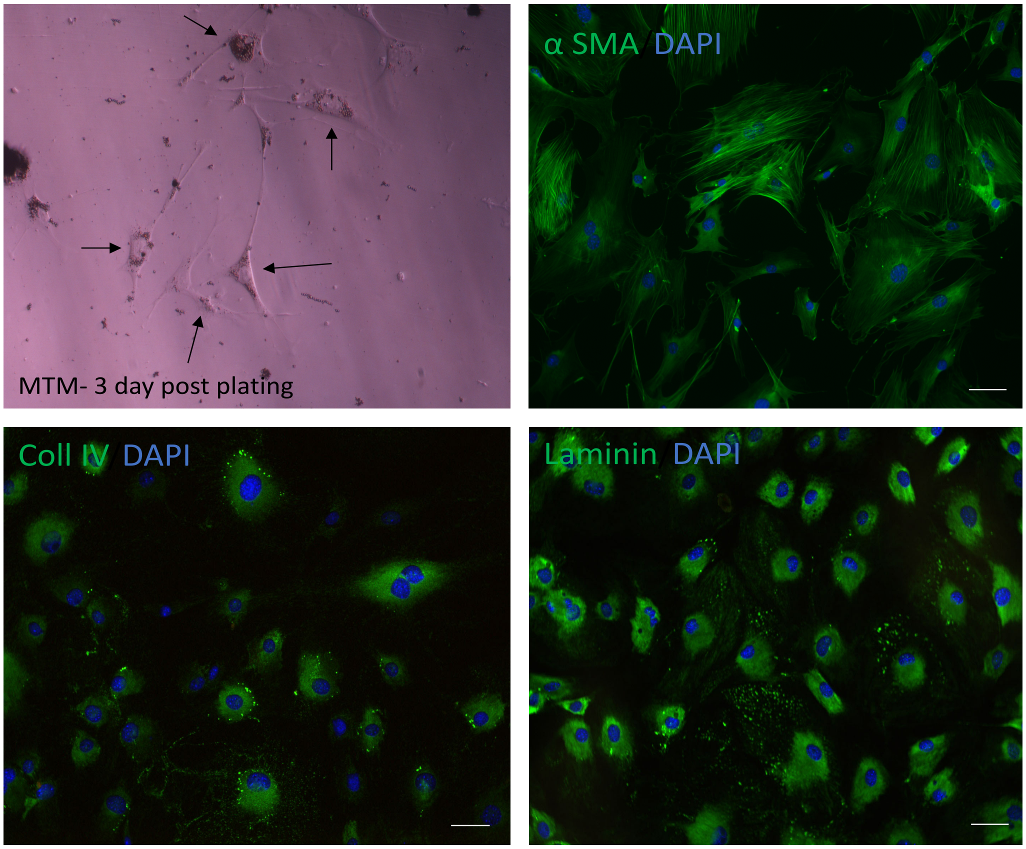

***Figure S1**: Characterization of MTM cells from C57BL/6J mice. Bright field image showing MTM cells in culture after 3 days of isolation (top left column). Black arrows showing MTM cells with engulfed magnetic beads. The expression of Coll IV (green) (bottom left column), α-SMA (green) (top right column), and laminin (green) (bottom right column) in MTM cells is shown by immunocytochemistry. Blue color represents DAPI staining showing cell nuclei. 20x magnification. Representative data for 3 experimental triplicates. Scale bar: 50 μm.*

Figure S2

A) B) C)

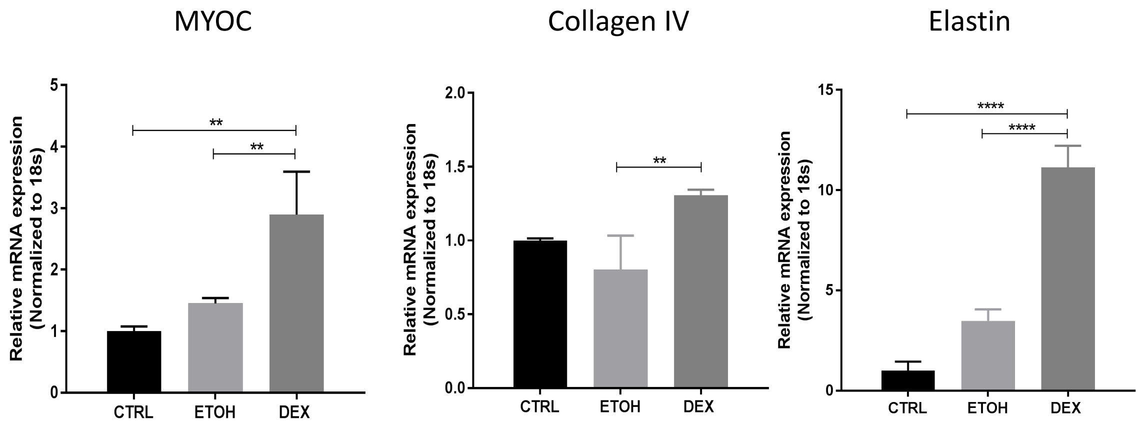

Figure S2*: DEX-induced changes in MTM cells is shown by qPCR. MTM cells were treated with DEX for 72 hours followed by qPCR analysis. DEX treatment increased expression of MYOC* ***(A)****, Collagen IV* ***(B)****, and Elastin* ***(C)*** *in MTM cells. CTRL represents control and ETOH represents ethanol treatment. Representative data for 3 experimental triplicates.*

Figure S3

A) B)

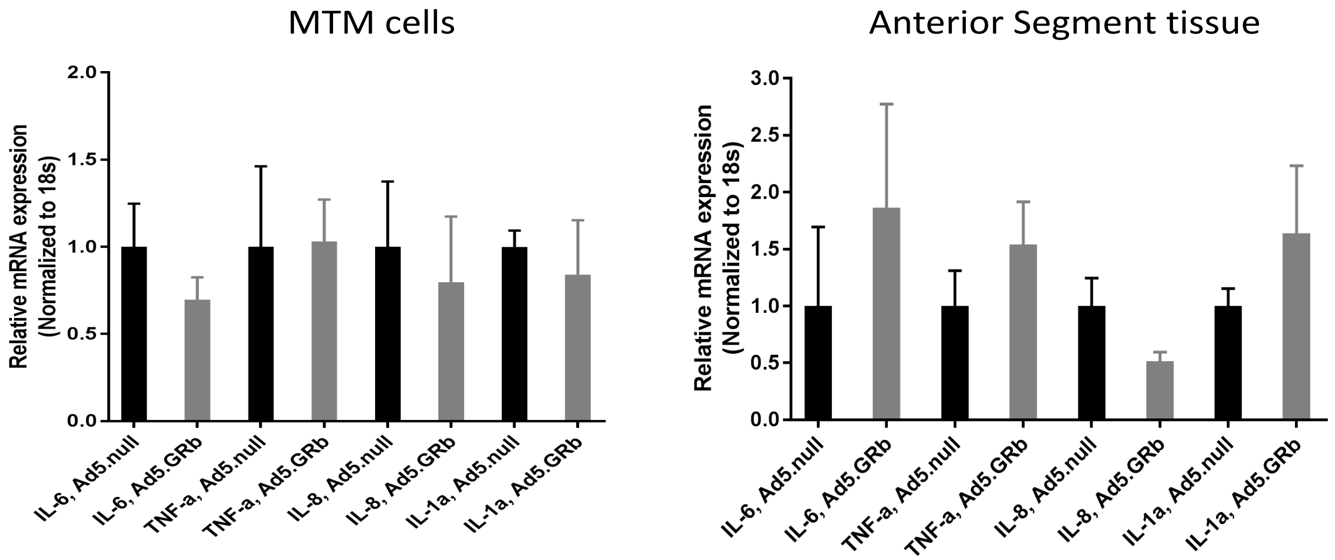

Figure S3*: Adenoviral vector (Ad5) transduction does not induce inflammatory response in TM. (A) Transduction of* Ad5.hGRβ *and Ad5.null vectors in cultured MTM cells* ***(A)*** *and anterior segment tissues* ***(B)*** *did not induce inflammatory response as shown by no change in expression inflammatory cytokines including IL-6, TNF-α, IL-8, and IL-1α. Data are presented as means ± SEM. Representative data for 3 experimental triplicates. Black bar shows Ad5.Null group and gray bar shows* Ad5.hGRβ treated group.

Full length Western blot images

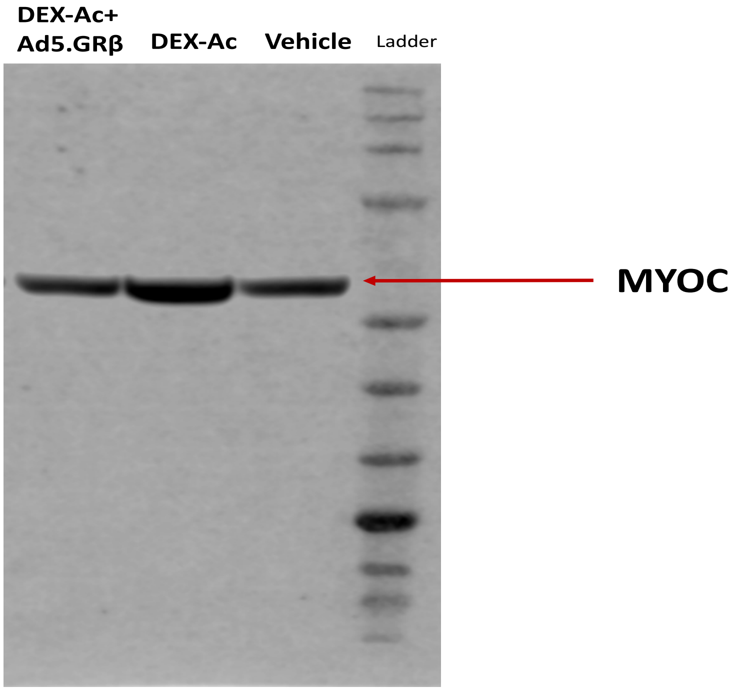

Figure S4*: Full length western blot for myocilin (MYOC)*

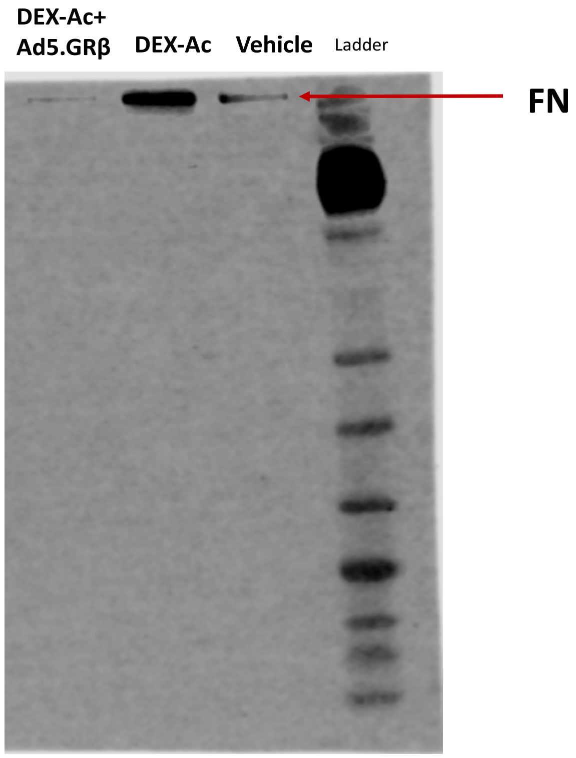

Figure S5: *Full length western blot for fibronectin (FN)*

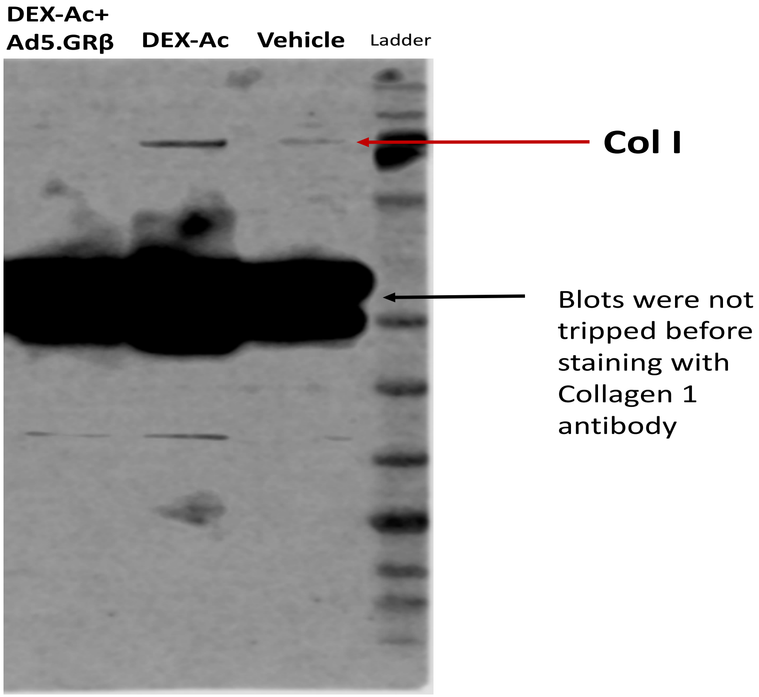

Figure S6: *Full length western blot for collagen I (Col I)*

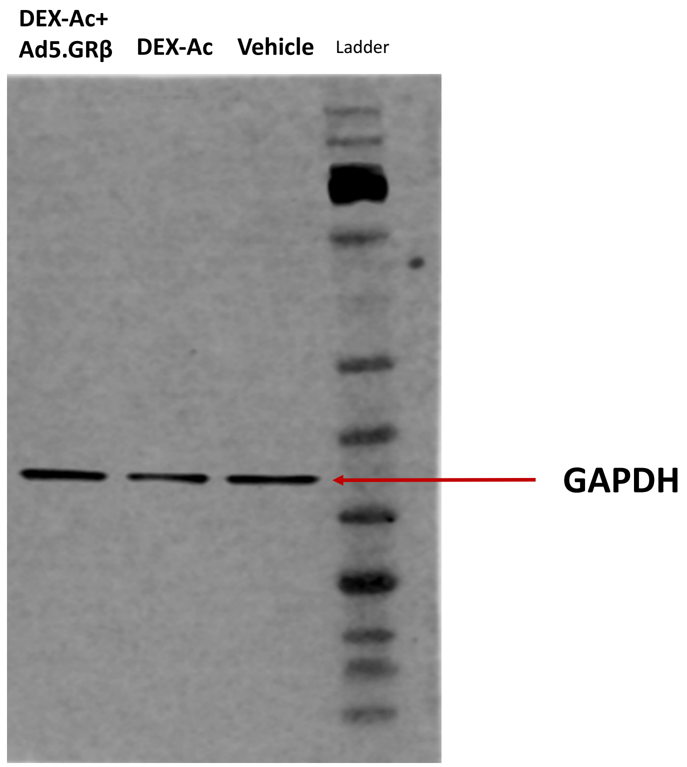

***Figure S7**: Full length western blot for GAPDH*

Table S1: List of primers

Gene	Forward primer (5'-3')	Reverse primer (5'-3')
18s	TTCGAACGTCTGCCCTATCAA	ATGGTAGGCACGGCGACTA
IL-6	AGTTGCCTTCTTGGGACTGA	TCCACGATTTCCCAGAGAAC
TNF-α	AGCCCCCAGTCTGTATCCTT	AGTCCTTGATGGTGGTGCAT
IL-8	TGAGGCAAGATCCCTCTGTT	CCAAGGTTTGAGTTCCCAGA
IL-1α	GGAGTCAACTCATTGGCGCT	GTGCAAGTCTCATGAAGTGAGC

Table S2: List of antibodies

Antibody	Dilution and catalog #
rabbit anti-collagen IV	1:100; catalog # L9393; Sigma-Aldrich Corporation, St. Louis, MO
rabbit anti-Laminin	1:100; catalog # SAB4500369; Sigma-Aldrich Corporation

mouse anti-a-smooth muscle actin (a-SMA) conjugated with FITC	1:100; catalog # F3777; Sigma-Aldrich Corporation
